# First record of the polychaete *Ficopomatus uschakovi* (Pillai, 1960) (Annelida, Serpulidae) in the Colombian Caribbean, South America

**DOI:** 10.3897/zookeys.371.5588

**Published:** 2014-01-17

**Authors:** Catalina Arteaga-Flórez, Vanessa Fernández-Rodríguez, Mario H. Londoño-Mesa

**Affiliations:** 1 Grupo de Limnología Básica y Experimental y Biología y Taxonomía Marina (LimnoBasE y Biotamar), Instituto de Biología, Facultad de Ciencias Exactas y Naturales, Universidad de Antioquia, A.A. 1226, Medellín, 05001000, Colombia

**Keywords:** Euryhaline, exotic species, polychaete, species distribution, taxonomy

## Abstract

The genus *Ficopomatus* (Serpulidae) consists of sessile, tubicolous polychaete annelid worms that may colonize a diversity of substrata, and tolerate considerable variations in salinity. Thus, members of this genus, including *Ficopomatus uschakovi*, in some cases are exotic and maybe invasive. The purpose of our research was to collect and identify marine organisms associated with the submerged roots of mangrove trees in the Gulf of Urabá, Colombian Caribbean, South America. Within the Gulf, there is a well-developed forest of the Red Mangrove, *Rhizophora mangle*, along the margins of El Uno Bay. We sampled the roots of *R. mangle* from five stations of the bay, and we identified specimens of *F. uschakovi* from each of those stations. *Ficopomatus uschakovi* was found to be more abundant in regions of the bay that exhibit the lowest salinity. Based on a morphological comparison of the present specimens with the original species description, revised descriptions, and other records from the Indo-West Pacific, Mexican Pacific, and Venezuelan and Brazilian Caribbean, we suggest that *F. uschakovi* has a broader geographical distribution. Furthermore, because of this broad distribution, and the observed tolerance for low salinity in our study, we also suggest that *F. uschakovi* is a euryhaline species. It is also likely that *F. uschakovi* will be found in other localities in the Gulf of Urabá, and in other regions of the Colombian Caribbean. Thus, this record extends the distribution of the species to the Colombian Caribbean, giving the species a continuous distribution across the northern coast of South America.

## Abstract (es)

El género *Ficopomatus*, familia Serpulidae, está compuesto por gusanos poliquetos tubícolas y sésiles que pueden colonizar gran cantidad de sustratos y tolerar variaciones considerables de salinidad. Así, los miembros de éste género, incluyendo *Ficopomatus uschakovi*, en algunos casos pueden ser considerados exóticos y tal vez invasores. El propósito de nuestra investigación fue recolectar e identificar los organismos marinos asociados a raíces sumergidas de árboles de mangle rojo, *Rhizophora mangle*, en el golfo de Urabá, Caribe colombiano, Suramérica. Dentro del golfo de Urabá, existe un bosque bien desarrollado de *R. mangle*, a lo largo de las márgenes de la bahía El Uno. Se tomaron muestras de mangle rojo en cinco estaciones y seidentificó a *F. uschakovi* para cada una de ellas. Esta especie exhibió mayor abundancia en zonas de la bahía con menor salinidad. Basados en la similitud morfológica que exhiben los ejemplares con la descripción original de la especie, descripciones revisadas e identificaciones reportadas en el oeste del Indopacífico, Pacífico mexicano y la zona Caribe de Venezuela y Brasil, sugerimos que *F. uschakovi* tiene una amplia distribución geográfica. Además, por esta amplia distribución y la tolerancia observada a bajas concentraciones salinas en nuestro estudio, sugerimos que *F. uschakovi* es una especie eurihalina. También es probable que *F. uschakovi* se pueda encontrar en otras localidades en el golfo de Urabá, y en otras regiones del Caribe colombiano. Este registro amplía la distribución de la especie al Caribe colombiano, lo cual demuestra una continuidad en la costa norte de Suramérica.

## Introduction

The family Serpulidae Rafinesque, 1815, includes a group of sedentary polychaetes that are easily recognizable by their calcareous tubes, with irregularly twisted or spiral growth, and by the complexity of their radiolar crown (Rouse and Pleijel 2001). However, other features, such as the shape of the operculum, the branchial crown, the form, type and position of chaetae along the collar, the thoracic membranes, as well as the thorax and abdomen, represent important taxonomic characters (Hove and Kupriyanova 2009). Some serpulids are solitary, but others such as *Hydroides* (Bastida-Zavala and Hove 2003), are gregarious, living in large groups or colonies that may cover the hard substrates of coral reefs, coastal lagoons, and the estuarine or brackish environments of tidal canals within mangrove forests (Bastida-Zavala and Salazar-Vallejo 2000). They are benthic, sessile organisms, and their tubes have been found attached to rocks, roots, wood, mollusc shells, dock structures, and boat hulls that facilitates their dispersal (Salgado-Barragán et al. 2004); few species have free tubes, unattached to any substratum, but living on a sandy bottom.

The genus *Ficopomatus* Southern, 1921 (subfamily Ficopomatinae Pillai, 1960) is characterized by having an opaque tube with or without keels, peristomes, and tabulae; a body that tapers in diameter from anterior to posterior; a conical or pear-shaped operculum inserted behind the left brachial lobe, uncovered or covered with either a chitinous, non-calcified endplate or with numerous chitinous spines in the distal tissue; the collar is non-lobed with “saw-edged” chaetae; the thorax may have free or fused thoracic membranes; there are seven chaetigers with limbate notochaetae, and six uncinigerous tori having uncini with 6 to 12 teeth; the abdomen contains numerous segments with capillary-toothed chaetae (Day 1967, Hove and Weerdenburg 1978, Hove and Kupriyanova 2009). The genus comprises six species: *Ficopomatus enigmaticus* (Fauvel, 1923), *Ficopomatus macrodon* Southern, 1921, *Ficopomatus miamiensis* (Treadwell, 1934), *Ficopomatus shenzhensis* Li, Wang & Deng, 2012, *Ficopomatus talehsapensis* Pillai, 2008 (Bastida-Zavala and García-Madrigal 2012) and *Ficopomatus uschakovi* (Pillai, 1960). According to Pillai (2008), *Ficopomatus uschakovi* should be included in *Neopomatus* Pillai, 1960 due to the presence of one pair of fused thoracic membranes; nevertheless, several authors do not accept this genus (Hove and Kupriyanova 2009, Bastida-Zavala and García-Madrigal 2012).

*Ficopomatus uschakovi* was described from Sri Lanka (Pillai 1960), and is widely distributed across the Indo-West Pacific (Hove and Weerdenburg 1978). It has been recorded in other regions, including the Mexican Pacific (Bastida-Zavala and García-Madrigal 2012), the Western Atlantic, in Paraiba, Brazil (de Assis et al. 2008), in Venezuela (Liñero-Arana and Díaz-Díaz 2012), and in the Eastern Atlantic, in the Gulf of Guinea (Hartmann-Schröder 1971). Therefore, *Ficopomatus uschakovi* is thought to be an exotic, and possibly, an invasive species.

According to the World Wildlife Fund for Nature (2012), exotic and invasive species are those foreign species that have been naturally or artificially (accidentally or intentionally) introduced to a region where they did not exist before, and once there, are adapted to their new environment. Liñero-Arana and Díaz-Díaz (2012) mentioned that the presence of invasive species in a location outside their current distribution might result from five recognized causes: First, transfer by fouling communities (fouling) attached to the outside of boat hulls; second, introduction of species for culturing, or as bait for fishing; third, exchange of species through waterways; fourth, release of species related to the pet industry; and fifth, expulsion of organisms from the ballast waters of ships. In order to better understand the mechanisms by which a particular species may be found outside of its natural range, it is necessary to examine each of these five causes and their potential influence where such species are positively identified. Further, it is critically important to determine the presence of invasive species that may threaten or even lead to the extinction of native species by competition or loss of habitat (Castro-Diez et al. 2004).

This study provides the first record of *Ficopomatus uschakovi* in the Gulf of Urabá, in a region that has been recently studied in the context of marine biodiversity. Additionally, our research provides a morphological comparison of *Ficopomatus uschakovi* with a sympatric species, *Ficopomatus miamiensis*, which may be competing for resources with the former, likely exotic, species within the Gulf.

## Materials and methods

**Study area.** The Gulf of Urabá is located on the northern coast of Colombia adjacent to the Isthmus of Panama, and is part of the Nica-Colombian continental shelf (Salazar-Vallejo 2000). The Gulf is part of the territory of Colombia’s Department of Antioquia, and it is considered the second largest estuary of the Colombian Caribbean, representing 4291 km^2^ of costal area, of which 650 km^2^ are estuaries, dominated by mangrove forests (CORPOURABA 2003). According to Bernal et al. (2005), the Gulf of Urabá is a semi-enclosed body of water, approximately 80 km long and 25 km wide, with an average depth of 25 m, and maximum depth of 60 m. The Gulf receives fresh water from the Atrato River in the west, and other smaller rivers that empty into its southern end. The northern end of the Gulf is open to the Caribbean Sea, which interacts with river inflows to generate estuarine circulation patterns, including substantial freshwater surface layering. Within the Gulf, a relatively large number of bays and inlets have been formed as a result of the high levels of suspended sediments carried in from surrounding rivers, which in turn supports the establishment and growth of *Rhizophora mangle* (Red Mangrove), *Laguncularia racemosa* (White Mangrove), *Avicennia germinans* (Black Mangrove) and fern forests.

**Figure 1.**
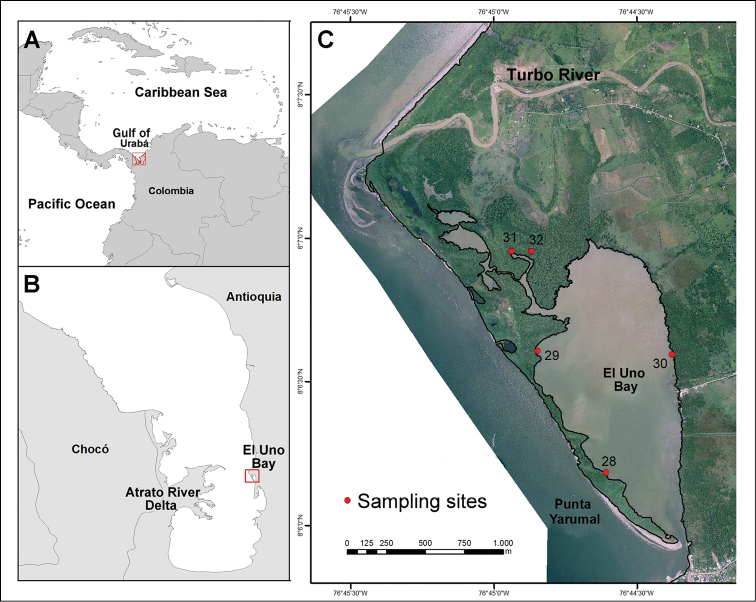
Study Area: **A** Colombia **B** Gulf of Urabá **C** El Uno Bay (Red points correspond to sampling sites).

El Uno Bay is located near the southeastern end of the Gulf, to the north of the municipality of Turbo (Fig. 1A–C). The bay is approximately 1.8 km long, 0.89 km wide, with an average depth of 1.0 m, and extends in a north-south direction. According to the mangrove system by García-Padilla and Palacio (2008), and our observations during this research, El Uno Bay is subdivided into the following zones: Northern zone with *Avicennia germinans* dominant in the inner region, and *Rhizophora mangle* in the outer region; northwestern fringe zone with well-developed *Laguncularia racemosa* trees and low numbers of *Rhizophora mangle* due to harvesting; eastern zone with *Avicennia germinans* dominant in the inner region, and young and mature trees of *Rhizophora mangle* in the outer region; southwestern fringe zone with sparse overall development dominated by *Rhizophora mangle* andsome *Avicennia germinans* trees; and the southern zone with a semi-enclosed circulation system, receiving high and low tidal water exchange, and without mangrove trees.

**Sampling.** El Uno Bay was sampled at five stations during August 2009 (Fig. 1C). Sampling was conducted according to the following collection process: (i) selection of the submerged roots of *Rhizophora mangle* trees not reaching the substrate, when possible; (ii) cutting and removal of 1–5 roots from different trees at each station, (iii) fixation of roots in a solution of 10% formalin in sea water.

In the laboratory, sampled roots of *Rhizophora mangle* were processed as follows: (i) formalin was removed by multiple freshwater exchanges through a 250 µm sieve to retain organisms; (ii) invertebrate and algal specimens (macroalgae, cyanobacteria) were selected and removed from roots; (iii) invertebrate specimens were preserved in 70% ethanol; macroalgae were preserved in 4% formaldehyde; (iv) invertebrates were separated into higher-level taxa (e.g. phylum, class, family), and (v) polychaetes were identified to levels of family, genus and species. The polychaete specimens were deposited in the Colección de Invertebrados Marinos, Universidad de Antioquia (CIMUA).

**Taxonomic analysis.** Four polychaete families were identified (Nereididae, Sabellidae, Serpulidae, Spionidae); however, only serpulids were considered for this research. For polychaete identification, we followed the dichotomous, taxonomic keys for genera prepared by Fauchald (1977), Bastida-Zavala and Salazar-Vallejo (2000) and Bastida-Zavala (2009). For identification of the serpulid polychaete species, *Ficopomatus uschakovi* and *Ficopomatus miamiensis*, we followed taxonomic keys and/or descriptions from Hove and Weerdenburg (1978), Bastida-Zavala (2009) and Bastida-Zavala and García-Madrigal (2012).

## Systematics

### Family Serpulidae Rafinesque, 1815
Subfamily Ficopomatinae Pillai, 1960
Genus *Ficopomatus* Southern, 1921

#### Ficopomatus uschakovi

(Pillai, 1960)

http://species-id.net/wiki/Ficopomatus_uschakovi

Figure 2A–J

**Figure 2.**
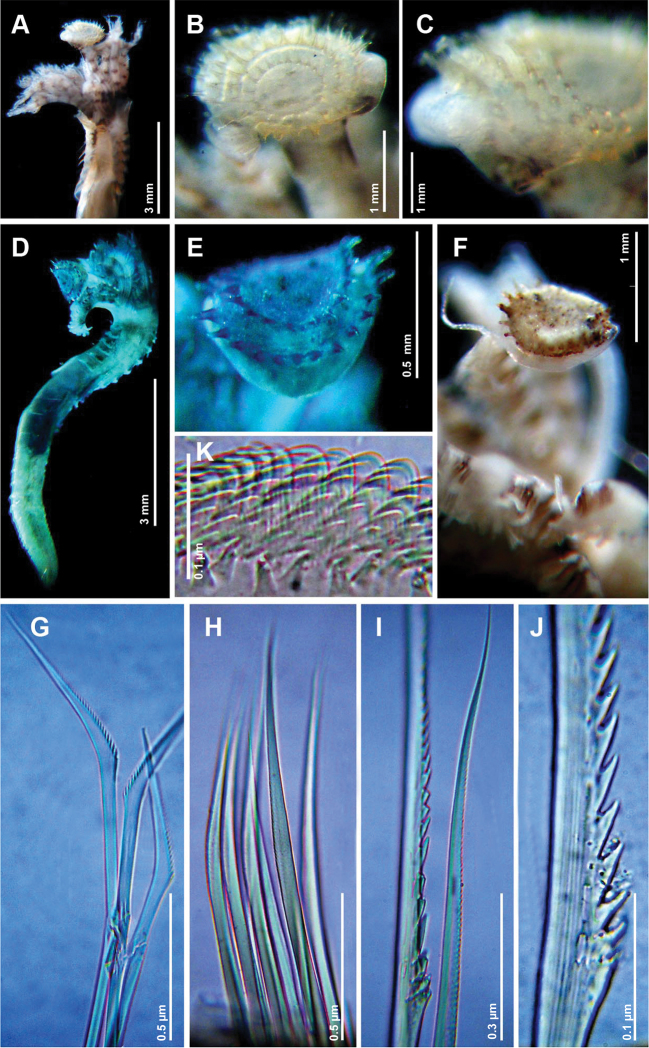
*Ficopomatus uschakovi* (Pillai, 1960). Specimen SERP 0031B: **A** Antero-dorsal view **B** Operculum in anterior view **C** Operculum in lateral view **G** Geniculate chaetae from the abdomen **H** Limbate chaetae from chaetiger 3 **I** Toothed and limbate chaetae from collar **J** Toothed chaetae, detail **K** Uncini from chaetiger 3. Specimen SERP 0033 in methyl green: **D** Complete specimen in dorsal view **E** Operculum in lateral view. Specimen 1 SERP 0034: **F** Operculum in lateral view.

Neopomatus uschakovi Pillai, 1960: 28–32, fig. 10H, 11A–H, 12A–H, plate I, fig. 1, 2; Hartman 1965: 80; Pillai 1965: 172: 1971: 118–123, 127, fig. 9G, 10; Hartmann-Schröder 1971: 7–27. fig. 2, 3, 5, 7b-d, 11–14; Zibrowius 1973: 64.
Mercierella enigmatica , (not Fauvel 1923, Mesnil and Fauvel 1939): Hove and Weerdenburg 1978: 109–110, presented several examples of incorrect use of this name.
Neopomatus uschakovi var. *lingayanensis*, Pillai 1965: 170–172, fig. 23A–I.
Neopomatus similis , Pillai 1960: 32–33, fig. 12 I–M, plate II, fig. 1; Hartman 1965: 80.
Neopomatus similis var. *rugosus*, Pillai 1960: 33–35, plate II, fig. 2; Hartman 1965: 80.
Ficopomatus uschakovi , Hove and Weerdenburg 1978: 109–113, fig. 2a–d, 3a, f–k, 4j–n, r, x–z, 5d; Bastida-Zavala 2009: 530, fig. 1K; Bastida-Zavala and García-Madrigal 2012: 48–52, fig. 1A–E, 2A–I.

##### Type locality.

Estuary of the Panadura River, Sri Lanka, Indian Ocean.

##### Material studied.

CIMUA POLY SERP 0031B (1), Punta Yarumal (8°6'10"N, 76°44'36"W), El Uno Bay; Sta. 28, Root 3; August 8, 2009; col. C. Arteaga-Flórez. CIMUA POLY SERP 0032 (1), Punta Yarumal, (8°6'34"N, 76°44'22"W), El Uno Bay, Sta. 29, Root 5, August 8, 2009, col. C. Arteaga-Flórez. CIMUA POLY SERP 0033 (1), El Faro (8°6'36"N, 76°44'50"W), El Uno Bay; Sta. 30, Root 5; August 8, 2009; col. C. Arteaga-Flórez. CIMUA POLY SERP 0034 (5), Ciénaga de las Mujeres (8°6'55"N, 76°44'51"W), El Uno Bay; Sta. 31, Root 1; August 8, 2009, col. C. Arteaga-Flórez. CIMUA POLY SERP 0035 (12), Ciénaga de las Mujeres (8°6'56"N, 76°44'56"W), El Uno Bay; Sta. 32, Root 5; August 8, 2009; col. C. Arteaga-Flórez.

##### Description.

Complete specimen: Irregularly curved calcareous tubes, forming agregations. Thorax colour, brown; tori colour dark brown; abdomen colour beige; dark brown medial line along the dorsum. Length, 5.0 mm; width, 0.6 mm, with 7 thoracic chaetigers and 33 abdominal chaetigers. Branchial crown colour beige, divided into two groups of radioles: six radioles on right side, and seven radioles on left side; each radiole with six transverse bands in a ring-like arrangement with wide dark brown color pattern; most basal ring wider and darker than the rest; ventral base of branchial crown black. Inter-radiolar membrane absent. Operculum spherical with radial symmetry (Fig. 2B, C, E, F), and with a sub-convex distal plate; four rows of transparent spines directed outward, with spines of the interior row approximately one-half the length of spines in the other rows; peduncle colour, beige with dark brown groove. Eyes absent. Collar with entire margin; thoracic membranes fused along the dorsum; six thoracic neuropodial tori. Chaetae from collar serrated, with one longitudinal line of teeth from the base to the apex (Fig. 2I, J), thorax with capillary chaetae (Fig. 2H), and abdomen with geniculate chaetae, proximal half serrated (Fig. 2G), and distal half smooth. Thoracic uncini saw-shaped with 6–7 teeth (Fig. 2K). Rounded pygidium with a midline incision.

##### Variations.

The specimens vary in length from 3 mm to 7.5 mm, in width from 0.5 mm to 0.8 mm. The number of chaetigers varies from 36 to 45. The number of transparent rows of spines from the operculum varies from 1 to 4. The number of transversal rings in the crown varies from 4 to 6.

##### Remarks.

*Ficopomatus uschakovi* was present in samples from the opening of El Uno Bay, in sympatry with *Ficopomatus miamiensis*, although *Ficopomatus uschakovi* was more abundant inside of El Uno Bay. Of these two serpulid species, only *Ficopomatus uschakovi* was found within the inner bay, which may indicate that this species has replaced *Ficopomatus miamiensis* in that region. Morphologically, *Ficopomatus uschakovi* differs from *Ficopomatus miamiensis* by the presence of a spherical operculum with 1–4 transparent spines in a radial arrangement, and dorsal fusion of the thoracic membranes. These characters represent important diagnostic features for recognizing *Ficopomatus uschakovi*, according to Hove and Weerdenburg (1978). Of the remaining taxonomic characters, the presence of seven thoracic chaetigers, and toothed collar chaetae, are generic characters. Distinctively, *Ficopomatus uschakovi* builds calcareous tubes with longitudinal keels, and well-formed rings, whereas *Ficopomatus miamiensis* builds smoother calcareous tubes that exhibit only very diffuse growth rings (Hove and Weerdenburg 1978).

Hove and Weerdenburg (1978) comment that *Ficopomatus uschakovi* has been commonly misidentified as *Ficopomatus enigmaticus*, because both species are able to live in brackish water. However, Hartmann-Schröder (1971) and Pillai (1971) clarified this confusion, emphasizing on the geographical separation of these species; *Ficopomatus enigmaticus* occurs in subtropical regions of Europe, while *Ficopomatus uschakovi*, originally recorded in the Indo-West Pacific region, was recently found in the Caribbean Sea and the Tropical Eastern Pacific. Nevertheless, according to Hove and Weerdenburg (1978), the most important features for splitting these two species are in terms of the morphology. While *Ficopomatus uschakovi* has an operculum with a sub-convex distal plate, *Ficopomatus enigmaticus* has a concave distal plate. Also, *Ficopomatus enigmaticus* sometimes has, dorsally, incomplete and irregular rows of spines, and may have one to three short radial accessories spines, while *Ficopomatus uschakovi* lack accessory spines and has complete and regular rows of spines in the operculum.

According to Hove and Weerdenburg (1978), *Ficopomatus uschakovi* has a distribution in the southern hemisphere, from India, through the Indian Ocean, to the Philippines and northern Australia. However, it has been recorded also from the Caribbean region, in Venezuela (Liñero-Arana and Díaz-Díaz 2012) and north-eastern Brazil (de Assis et al. 2008); and from the tropical Eastern Pacific, in Mexico (Bastida-Zavala and García-Madrigal 2012).

We consider *Ficopomatus uschakovi* as an exotic species in the Colombian Caribbean. It is likely that this species may have been transported to the Gulf of Urabá from the Indo-Pacific attached to hulls of ships crossing from the Pacific Ocean to Caribbean Sea through Panama Canal, or from the Eastern coast of Africa to the Caribbean. Once in the Gulf, the species migrated to El Uno Bay, which is very close to the place where the ships are charged, aided by the tidal currents. Also, in support that *Ficopomatus uschakovi* is an exotic species: during 2009 the species was found only in El Uno Bay, southern Gulf of Urabá; but, during 2012, this species was found also to the north of El Uno Bay, where it was not found before. This means that the distribution of the species has spread along the eastern coast of the Gulf in a South-North direction. This study provides the first record of *Ficopomatus uschakovi* in Colombia. However, many localities of the northern Colombian Caribbean lack information on the distribution of *Ficopomatus uschakovi*, which limits our understanding of its distribution in the southern Caribbean. Further distributional data will be presented in a forthcoming paper on the biogeography of the species.

Finally, specimens of *Ficopomatus uschakovi* were found on mangrove roots in association with specimens of other species belonging to the families Nereididae (*Neanthes succinea*, *Stenoninereis tecolutlensis* and *Namalycastis* sp. 1), Sabellidae (*Demonax lacunosus*), and Spionidae (*Boccardiella* sp.). Future research should include formal descriptions of these other species, and an assessment of their respective distribution patterns. Physical-chemical conditions found within the Bay during the sampling period are provided in Table 1.

**Table 1.** Physical-chemical conditions found in El Uno Bay, during the sampling period. Abbreviations: O_2_ oxygen; T Temperature; TDS dissolved solids; Sal Salinity; Cond Conductivity.

Locality	Site	Station	[O_2_] (mg/L)	O_2_ (%)	T (°C)	pH	TDS (ppt)	Sal (ppt)	Cond. (mS)	Specific cond. (mS)
El Uno Bay	Punta Yarumal	28	4,18	61	30,4	7,13	> 2000	9,2	17,41	13,88
El Uno Bay	Punta Yarumal	29	5,16	66,6	29,8	7,22	> 2000	9	16,87	15,48
El Uno Bay	El Faro	30	4,33	69,7	28,6	7,53	> 2000	9,2	14,85	15,57
El Uno Bay	Ciénaga de las Mujeres	31	2,12	31,2	27,5	7,3	> 2000	0,4	0,16	0,09
El Uno Bay	Ciénaga de las Mujeres	32	0,55	7,6	26,4	7,35	> 2000	1,3	13,54	11,72

##### Distribution.

Indian Ocean from the Eastern coast of Africa to Australia; Eastern Pacific in Southern Mexico; Western Atlantic, in the Colombian, Venezuelan and Brazilian Caribbean coasts. Eastern Atlantic, in the Gulf of Guinea.

## Supplementary Material

XML Treatment for Ficopomatus uschakovi
